# The ethics of using virtual assistants to help people in vulnerable positions access care

**DOI:** 10.1136/jme-2024-110464

**Published:** 2025-02-17

**Authors:** Steven R Kraaijeveld, Hanneke van Heijster, Nadine Bol, Kirsten E Bevelander

**Affiliations:** 1 Department of Ethics, Law & Medical Humanities, Amsterdam UMC, Amsterdam, Netherlands; 2 Department of Communication and Cognition, Tilburg University, Tilburg, Netherlands; 3 Department of Primary and Community Care, Radboud University Nijmegen Medical Centre, Nijmegen, Netherlands

**Keywords:** Ethics, Quality of Health Care

## Abstract

People in vulnerable positions who need support in their daily lives often face challenges in receiving timely access to care; for instance, due to disabilities or individual and situational vulnerabilities. There has been an increasing turn to technology-mediated ways to improve access to care, which has raised ethical questions about the appropriateness and inclusiveness of digitalising care requests. Specifically, for people in vulnerable positions, digitalisation is meant to facilitate requests for access to healthcare resources and to simplify the process of navigating the healthcare system. In a multidisciplinary research project, we examined the use and value of a ‘sensitive’ virtual assistant that can accommodate different needs of target groups through inclusive design, adaptive technology and artificial intelligence. This paper presents empirical findings from focus groups with care recipients and caregivers about the sensitive virtual assistant and relates the findings to five larger ethical issues associated with the use of virtual assistants in healthcare settings and care practices more generally. It highlights the risk that, even with the inclusion of target groups in the design of digitalised care assistants, some people may benefit significantly less than others.

## Introduction

 People in vulnerable positions who need support in their daily lives—for example, due to disabilities or individual and situational vulnerabilities—often face challenges in gaining timely access to care.^1^ There has been an increasing turn towards technology-mediated ways to provide better and more timely care, particularly given the rising costs of health and social care in many countries around the world.^2^,^4^

One approach has been to digitalise different aspects of health and social care; for instance, through artificial intelligence (AI), eHealth or mHealth technologies.^5^,^7^ Digitalisation is meant to improve access to care by making it more readily available, especially when in-person interactions are not feasible. Additionally, digitalisation aims to simplify the process of receiving care.^8^ One example of a digital health tool that can improve access to care and potentially simplify the process of receiving it is a ‘virtual assistant’—a tool that ‘accepts natural language as input and generates natural language as output, engaging in a conversation with a user,’^9^ such as a chatbot or artificial conversational agent.^10^ Virtual assistants, which can provide automated and standardised responses in a conversational manner to address various informational and motivational needs of care recipients,^11^ are increasingly implemented across various healthcare settings and for different target groups.^12^,^14^ At the same time, the rise of such digital health tools raises important ethical questions about when, how and for whom they ought to be used.^15^,^17^

Following these developments, a recent multidisciplinary research project examined the design and value of a ‘sensitive’ virtual assistant (SVA)^1^ in the form of a chatbot to accommodate the different needs of people in vulnerable positions through adaptive and inclusive design and AI.^18^ The SVA is considered ‘sensitive’ because it was designed to respond to particular needs and capabilities of the target group in question, namely people in vulnerable positions who need long-term care and support in their daily lives, such as individuals with cognitive impairments. In the SVA project, ‘citizen scientists’—coresearchers with cognitive impairments, including intellectual disabilities or autism spectrum disorder (ASD)—actively participated as team members throughout the entire duration of the project. For example, they comoderated and analysed focus group sessions with both care recipients and care givers.^15^ Including target populations that potentially benefit from interventions is important not only to ensure that interventions address their specific needs and concerns, but it also allows them to actively participate and shape the research that directly concerns them.^19 20^

So far, there has been little engagement with ethical questions about developing and using this technology specifically for people in vulnerable positions. We address this gap with an empirically informed ethical analysis. Our approach is grounded in empirical ethics, which combines philosophy with empirical research.^21^ Specifically, we present a secondary analysis of focus group data collected in the ongoing SVA research project^18^ and relate the findings to larger ethical issues surrounding digitalisation and access to care for people in vulnerable positions.

## Methods

### Study design

This study involved a secondary analysis of data previously collected (September 2022–February 2023) through focus groups with care recipients (ie, people in a vulnerable position) and caregivers.^18^ The aim of the focus groups and the initial analysis was to understand the daily care needs of individuals in these groups and their experiences in obtaining (information about) care and support. Caregivers were also included because they are closely involved in care recipients’ lives and can, therefore, provide additional insights into their care needs and experiences. To ensure that care recipients and caregivers could express their perspectives independently, each participated in separate focus groups.^2^ Focus groups can identify a variety of experiences and perspectives at once,^22^ which was important given the diversity of care recipients and types of caregivers. In the secondary analysis reported in this paper, the focus group data were analysed through a different lens, namely by focusing on participants’ perspectives specifically about the SVA. These perspectives were beyond the scope of the initial analysis, yet the authors opted to separately analyse them as they revealed considerations about the SVA that appeared to be important for a broader ethical reflection.

### Participants and data collection

The focus groups comprised 23 care recipients and 13 caregivers (50% female), age range 23–83 years (average: 47.8 years) with primarily Dutch background (92%). The number of participants in the different focus groups is provided in table 1.

**Table 1:** Number of participants in the different focus groups

Focus group	Participant group	N
1	Care recipients	6
2	Care recipients	4
3	Care recipients	2
4	Care recipients	4
5	Care recipients	4
6	Care recipients	3
7	Caregivers	4
8	Caregivers	4
9 (individual interview)	Caregivers	1
10	Caregivers	4

Most participants were recruited through the networks of the partners in the SVA project,^18^ including an academic collaborative for health of people with a disability as well as a foundation for social care support. Care recipients had various diagnoses, including intellectual disability, ASD, acquired brain injury or mental disorder. Both professional caregivers (n=9) and informal caregivers (n=4) participated. Professional caregivers were, for instance, a client advisor (ie, social care worker), an individual supervisor or a psychologist. Informal caregivers were people taking care of a family member (eg, mother, daughter and partner).

### Data analysis

In the initial study, focus groups were audiorecorded and transcribed verbatim.^18^ From the transcripts, the parts about the initial reactions to the SVA were used for the secondary analysis of this study. The secondary analysis consisted of thematic content analysis to identify recurrent concepts that summarised the range of experiences expressed by participants.^22^ This method was considered most suitable due to the exploratory character of this study. An inductive approach was used to explore ethical considerations of a virtual assistant, allowing the study to stay close to the data without fitting to an existing theoretical framework.^23^

A three-step coding process of open, axial and selective coding was applied.^24^ First, transcripts were read by HvH, NB, and KEB to make an initial coding list. During the entire coding process, codes were discussed between HvH and KEB and subsequently merged if they were about a similar topic (eg, ‘wanting to be seen by a human’ and ‘non-verbal communication is important’). Next, the codes were clustered into subthemes, leading to three main themes. The (sub)themes were discussed among all authors to secure validity (see online supplemental appendix 1 for the coding tree). Three main themes and seven subthemes emerged (see table 2 for an overview).

**Table 2:** Overview of three identified themes with respective subthemes and examples from participants

Theme	Subtheme	Example
Lower threshold for some care requests.	1.1. Helpful in referrals.	“I can ask questions about health: 'I have pain in my kidneys,' whom should I contact?* –Care recipient, FG9
	1.2. Reducing mental barriers for care requests.	“The psychologist is not always available, but I just want to talk with someone. If I can talk to a robot, I might feel lighter.” –Care recipient, FG9 “I did phone for help at a certain point, but it was not an easy step to take.(…)Maybe it [chatbot] could have been an easier way, at least for some people.” –Care recipient, FG4
Unsuitability for some care requests.	2.1. Unsuitability for complex care requests.	“It may be useful for simple questions, but when it becomes a bit more complex you will get stuck.” –Informal caregiver, FG 10
	2.2. Lack of authenticity.	“A chatbot cannot 'listen' to me.” –Care recipient, FG 9 “I wouldn't use it(…)(I)t’s unpersonal.(…)You just receive standard messages.” –Care recipient, FG 5 “I just need to see a person, otherwise I cannot use it.” –Care recipient, FG 2
	2.3. Privacy and reliability of sensitive information.	“Imagine it’s about a sensitive topic, then it gets more complicated with privacy.” –Care recipient, FG 5. “If it gives you an answer, how do you know where it comes from and that it is right?” –Informal caregiver, FG10
Unequal accessibility.	3.1. Difficulty in formulating questions.	“I honestly think that formulating your request for help, is a request for help in itself. " –Care recipient, FG5 "If I need a digital answer, I don't know what I should ask, what I can ask… It will be an endless search.” –Care recipient, FG2
	3.2. Lack of communication and digital skills.	“I have aphasia, so I wouldn't be able to write down a question in a clear manner.” –Care recipient, FG2 “Make no mistake, for many care recipients these types of technologies are very difficult to use.” –Informal caregiver, FG10

* All translations from Dutch to English by HvH.

## Results

The first theme centres on the SVA’s perceived advantage: its potential to provide a low threshold to requests for care. The other two themes disclose concerns that participants had about the use of the SVA for care and support; specifically, regarding its suitability for receiving care, and its accessibility for a wide variety of individuals with diverse abilities and needs. In what follows, we discuss each theme and respective subthemes. Quotations for each theme are provided in table 2.

### Lower threshold for some care requests

The SVA was considered to be promising in providing a relatively low-threshold way to ask for help by (1) referring people to an appropriate place or person and (2) raising fewer ‘mental barriers’ for care and support requests.

#### Helpful in referrals

When care recipients wanted to find a specific form of support, they used search engines as a starting point. The SVA was expected to partly function as a search engine while providing more direction. For instance, the SVA might provide a list of relevant care-related topics, thus simplifying the process of formulating questions. By offering suggestions, the SVA could steer care recipients in the right direction.

Examples of care-related topics that participants mentioned were ‘domestic help’, ‘medical aides’, ‘finances’ or ‘work’. Yet, they also mentioned that, ideally, the SVA would refer them to a human being after having selected the relevant care topic. Furthermore, consensus emerged that the number of topics should be limited in order to avoid overwhelming care recipients with information.

#### Reducing mental barriers for care requests

The SVA was considered to be a potentially low-threshold first step towards seeking care due to its promise of reducing psychological and emotional barriers (like shame and insecurity) when reaching out for care and support. Care professionals stated that shame and fear of calling on the telephone could prevent care recipients from seeking support. For example, during an episode of depression, the barrier to telephone another person can be high. The SVA may be an easier way to reach out for help in such circumstances.

The SVA was also perceived as being a promising conversational partner; for instance, in the absence of human conversational partners, or when in need of a judgement-free and judgment-free place to share things and to potentially ease emotional and psychological burdens.

### Unsuitability for some care requests

Regarding the suitability of the SVA, concerns were raised that it may not be suited to answer some questions about care or to provide the required support. Three reasons were mentioned, including (1) the complexity of questions in care and support, (2) the importance of authenticity in care relations and (3) issues concerning privacy and the reliability of information.

#### Unsuitability for complex care

Care recipients perceived themselves as having complex care questions about their specific, often multifaceted situations. For example, questions and requests for care may be about problems across various life domains or in relation to multiple diagnoses. Participants indicated that the SVA would only be able to give answers to relatively simple and standard questions, which might not accurately reflect the complexity of their daily challenges.

Both care recipients and professionals were sceptical about whether the SVA would be able to provide sufficient support. After all, even human professionals struggle to answer certain questions, such as those related to the complexity of the Dutch benefit system or to different regional regulations.

Furthermore, bad prior experiences with online chatbots contributed to scepticism about and resistance to the SVA. Previous encounters had led to frustration with and negative perceptions of virtual assistants (see table 2 for examples). Participants expressed the fear that the SVA would be unable to understand their questions or provide much-needed answers.

#### Lack of authenticity

Worries were also expressed about the lack of ‘humanness’ in interactions with the SVA. Participants emphasised that in (health)care interactions, they valued contact with a human caregiver and that the SVA could not replace or imitate those human encounters. A real person was perceived as being more understanding than a virtual assistant, especially because a caregiver notices subtle behaviours or feelings and uses non-verbal communication. For example, in video-mediated conversations with care professionals during the COVID-19 pandemic, participants found it more difficult to see the bodily expressions of care professionals. They expected this to be worse with the SVA. Participants also expected that the SVA would be ‘impersonal’ due to its ‘standard’ and too narrowly predefined answers.

However, it was mentioned that the SVA might be able to give more specific answers because of emerging technologies like ChatGPT, which participants saw as an improvement over older (non-AI-generated) kinds of chatbots.

#### Privacy and reliability of sensitive information

There were concerns related specifically to the sensitivity of the care-related information that would be shared with the SVA. Regarding privacy, it was unclear to participants where and with whom the users’ input would end up. Participants generally worried about sharing sensitive information about diagnoses, personal health issues, and other intimate topics with the SVA.

The reliability of information from the SVA was also questioned by care recipients. Whether users could trust the information they would receive from the SVA was a particular concern, given the sensitive nature of the information and the significant potential consequences that a lack of reliability would have.

### Unequal accessibility

Participants expressed concerns related to another theme, namely about the accessibility of the SVA. They doubted whether the SVA would be able to help everyone because they expected challenges with (1) the formulation of questions and (2) the required communication skills to engage with it. Given the diverse needs and abilities of its potential users, they expected that not everyone would be able to benefit equally from the SVA.

#### Difficulty in formulating questions

When considering the SVA for care-related questions, participants foresaw difficulties in the formulation of suitable questions. Based on previous experiences with digital assistants, they expected the SVA to generate short and direct responses. In conversations with human care providers, or while performing internet searches, care recipients often struggled to formulate apt questions. This was expected to be even more difficult in conversations with the SVA.

Furthermore, care professionals mentioned that it is common for people to approach them with a question about a specific topic, only for it to become apparent during conversation that another topic needs to be addressed first. For instance, people might ask for help with tax issues, while what they actually need is support with debt. Formulating a request for help can therefore require help in and of itself; which the SVA, presumably, cannot do or is much less able to do than a human being.

#### Lack of communication and digital skills

Besides difficulties in formulating questions, participants expected challenges with the required communication skills necessary to properly engage with the SVA. Transferring a question into writing was mentioned as a major challenge. Care recipients with aphasia, for instance, expressed that writing was a very difficult task for them in their daily lives; they expected this to be no different when using the SVA. The expectations of alternatives for written communication in the SVA, such as speech functionality, were low. Similarly, care professionals mentioned that verbal communication is much more suitable for most care recipients than written communication. Informal caregivers also expressed more general doubts about the capabilities of some care recipients to manage technological tools such as the SVA.

## Discussion and ethical implications

The findings from the focus groups demonstrate that, although the SVA was regarded as a promising development for low-threshold access to care, there were concerns about its suitability and accessibility for all care recipients. We will now discuss five ethical implications of the findings and relate them to larger ethical discussions about the use of digital technologies for people in vulnerable positions and within healthcare more generally.

First, human contact was greatly valued by participants, as evidenced by the preference to be helped by human beings and the concerns about a lack of ‘authenticity’ in encounters with the SVA. By authenticity, participants seemed to mean something like ‘real’ (ie, personal) human contact, a way of being fully seen; a lack of which might raise the risk of dehumanisation—that is, a loss of human presence and, perhaps, of not being treated as fully human through having one’s concerns relegated to machines. This raises ethical questions about whether or to what extent the human element of care ought to be supplemented by technology for people in vulnerable positions. Technology can augment, but not replace, patient care.^25 26^ This idea is underlined by the finding that human caregivers were perceived as being better able to understand care recipients and requests than the SVA; for instance, by being attentive to aspects of communication that are difficult to fully digitalise and/or mimic by generative AI. There were concerns about nonverbal communication, which participants considered to be vital to being properly understood, and which is known to be an important part of physician–patient interactions^27^ that cannot be fully simulated by a chatbot.^3^ The recognition that formulating a question is in itself an act that often requires assistance is relevant, because non-verbal communication—understanding subtext, implicit requests and so on—is a human and professional skill not presently replicable by a virtual tool. Human assistance must therefore remain available for those less able to fully articulate their needs.

Yet, the results also show that an SVA could be a low-threshold way to ask for help, not only due to the nature of certain kinds of basic care requests, but also because facing another person can in itself form a barrier to receiving assistance. The SVA may be easier to interact with in some cases and may empower patients by giving them access to tailored information and responses that could offer them greater insight into their (health) situation and prepare them for interactions with healthcare workers.^28^ At the same time, to truly empower patients with knowledge, ‘effective responses must offer more than simply the presentation of correct facts.’^29^^(p.161)^ Given that complex questions were perceived as challenging and uncomfortable to ask a virtual assistant, empowerment cannot simply be assumed. We also cannot take for granted that interactions with the SVA will always be wholly benign and that the information provided will always be correct. Given the known problems with generative AI (eg, biases, manipulations and hallucinations),^30^ there are serious risks of harm to users that must be balanced against expected benefits. Interactions with AI-powered technologies carry greater risks when more pressing and intimate information about health is shared.^31^ Problematic attachment to and dependency on the technology, particularly if it is always available and ‘acts’ like a human conversational partner, is another risk. Patient safety must be foregrounded especially for individuals who are in vulnerable positions. Whether and how virtual assistants can ultimately empower care recipients is an open question that requires further research and reflection.

Second, there were concerns about privacy and data use, which constitute a major ethical problem for digitalisation more generally and for patient data in particular.^32^ The SVA needs to process highly sensitive health data and other personal information to offer tailored feedback to its users. How and by whom such data will be accessed and stored are long-standing ethical questions in bioethics^28^ that remain relevant here. If privacy is strictly maintained by researchers at universities and academic hospitals, this may alleviate some of the more pressing concerns. However, in practice, sharing patient information with private companies and third parties is a real possibility and persistent ethical risk.^33^ Furthermore, if the SVA and similar technologies are to be powered by software owned by Big Tech corporations (eg, in the case of AI-generated responses), there is an additional risk that such companies gain undue influence and impose their own values and incentives (eg, profit maximisation) that are not congruent with, and even run counter to, long-standing values in healthcare (eg, trust).^34^ Ethically acceptable privacy regulations and data sharing agreements must be in place as a condition for implementing digital tools like the SVA.

Furthermore, it may be difficult to fully inform users in vulnerable positions and thus to obtain their informed consent for data use. Health care institutions, workers and researchers who wish to implement virtual assistants have a moral duty to keep this in mind and to adjust information relevant to informed consent to individual levels of comprehension (eg, by using images rather than text).^4^ Explicit compliance with regulations such as the General Data Protection Regulation (GDPR) is clearly important as a minimum standard, which is an issue previously raised in relation to smart devices for people in vulnerable positions more generally.^35^

Relatedly, there are ethical questions about ownership and responsibility for the proper functioning and specific content of the virtual assistant, especially in cases where harm might be caused. How will the functioning of the SVA be monitored? If the SVA provides incorrect or inappropriate information, it needs to be clear who is morally responsible and who is accountable for correcting it. The SVA needs to be trustworthy for patients in order for them to comfortably and safely use the technology.^36^ If generative AI is going to be incorporated, then the myriad problems that have already been identified with it for the general population (eg, racist and sexist responses, hallucinations, manipulation) are only bound to have more severe implications for people in vulnerable positions.^37 38^ As a precondition, the AI models used by the SVA should be thoroughly vetted and adjusted to relatively narrow goals that meet the specific care needs of target groups.

Third, there is the risk of excluding people with reduced means of communication, which raises issues of justice and fair distribution of (access to) healthcare resources. Given the often-large variations in people’s communication and digital skills, people with a reduced capacity for verbal communication will not benefit as much—or will even be hindered—by text-based virtual assistants.^39^ Care recipients and professionals expressed fears that one would need to be able to read and write well to be able to use the SVA. When speech technology was explained, some participants responded with scepticism about its effectiveness, especially for people with aphasia. The use of generative AI may ease some of the burden of being precise in one’s written language—and speech technology may facilitate matters—thus ameliorating some of these concerns. Still, a major ethical risk is that not all people in vulnerable positions will benefit equally from the implementation of the SVA—and some may even face increased difficulties in receiving the necessary care, should the technology come to replace care that is currently provided by human beings. This would have the highly undesirable result of producing and exacerbate inequalities in access to care. Again, the option to request human care must remain.

Fourth, consulting and/or collaborating with target populations in the design of a virtual assistant—or in digitalised healthcare more generally—may not be sufficient to ensure that implementing the resulting tool is ethical. The most vulnerable people are already at a more general risk of being left behind in the so-called digital healthcare revolution.^40^ Even though inclusive design can enhance the accessibility of technology,^41^ there may be a naïve expectation that design outcomes are necessarily morally acceptable and/or beneficial for all those included.^42^ What our findings suggest is that this is not always true. Even when representatives from the target populations are actively included in designing a digital product like the SVA, some may still face difficulties and thus be left behind. Careful work still has to be done to examine the specific implications of these virtual care technologies for all members of vulnerable groups, in order to ensure that a majority of users who are happy to use digital tools like the SVA does not mask a struggling subgroup. No digital tool is bound to function ideally for everyone all the time. From an ethical perspective, it is crucial to consider those who are likeliest to be left behind.

Finally, there is a need to balance the expected gains from digitalised care against known and foreseeable risks. All things considered, will target groups truly benefit from using this technology compared with human-centred care? Of course, human care may not always be feasible, and technological alternatives may significantly reduce healthcare costs and offset some of the negative effects of shortages in healthcare personnel. The expectation that digital tools like the SVA could help answer relatively simple questions and decrease barriers related to shame, for instance, is consistent with previous research.^43^ Yet, as participants pointed out, asking a question is not always easy. Some people will need help formulating a question even before asking the SVA. What will be the value of the SVA for them—compared, for instance, to using an online search engine? Even if the SVA cannot replace human contact, it may feel more like a conversational partner and could provide more tailored feedback than a search engine. Still, frustration about the (mal)functioning of the SVA might problematically lead some to turn away from seeking care altogether. Assuaging these problems will partly depend on how successfully developers of these digital tools can accommodate the needs of target groups. Providing human contact as an option remains essential.

One limitation of the present study is that participants were not explicitly asked about their (ethical) concerns with the SVA. However, the fact that doubts were spontaneously raised by participants—including about the ethical issue of equal accessibility—clearly means that these were prominent concerns. Future research should explore these issues more directly and in greater detail.

## Conclusion

While the SVA for people in vulnerable positions shows promise in providing a low-threshold means to access care, it also raises ethical questions. Individuals who are at risk of benefiting least from the technology need to be considered not only in the design of digital tools like the SVA, but also and especially during implementation. Continued engagement with target groups and careful ethical analysis are needed if we are to make wider use of such technologies in healthcare.

## Supplementary material

10.1136/jme-2024-110464 online supplemental appendix 1

## Data Availability

Data are available on reasonable request.

## Footnotes

‘Sensitive’ in this context means that the virtual assistant uses artificial intelligence to recognise different types of users and adapts the way that it interacts with users accordingly.

As such, the focus groups were not conducted between caregiving dyads.

It should be noted that, in the future, so-called ‘deepfakes’ (hyper-realistic artificial audiovisual images) might augment AI-powered chatbots, leading to AI-generated representations of persons (like doctors or nurses) that might be able to simulate at least some forms of non-verbal communication.

It should be noted that providing users with too much information can be overwhelming and potentially discouraging, making it counterproductive rather than helpful. A balance needs to be struck so that users are sufficiently and meaningfully informed—against a background, of course, of trustworthy practices.

## References

[1] Bol N, Strycharz J, Helberger N (2020). Vulnerability in a tracked society: Combining tracking and survey data to understand who gets targeted with what content. New Media & Society.

[2] Bodenheimer T (2005). High and rising health care costs. Part 1: seeking an explanation. Ann Intern Med.

[3] Berg M, Heijink R, Zwakhals L (2010). Health care performance in the Netherlands: Easy access, varying quality, rising cost. Eurohealth (Lond).

[4] Erixon F, van der Marel E (2011). What Is Driving the Rise in Health Care Expenditures? An Inquiry into the Nature and Causes of the Cost Disease.

[5] De Raeve P, Gomez S, Hughes P (2017). Enhancing the provision of health and social care in Europe through eHealth. Int Nurs Rev.

[6] Siala H, Wang Y (2022). SHIFTing artificial intelligence to be responsible in healthcare: A systematic review. Soc Sci Med.

[7] Sax M, Helberger N, Bol N (2018). Health as a Means Towards Profitable Ends: mHealth Apps, User Autonomy, and Unfair Commercial Practices. *J Consum Policy*.

[8] Menvielle L, Audrian-Pontevia A-F (2017). The Digitalization of Healthcare: New Challenges and Opportunities.

[9] Araujo T, Bol N (2024). From speaking like a person to being personal: The effects of personalized, regular interactions with conversational agents. Computers in Human Behavior: Artificial Humans.

[10] Sezgin E, Huang Y, Ramtekkar U (2020). Readiness for voice assistants to support healthcare delivery during a health crisis and pandemic. NPJ Digit Med.

[11] Lisetti C, Amini R, Yasavur U (2015). Now All Together: Overview of Virtual Health Assistants Emulating Face-to-Face Health Interview Experience. *Künstl Intell*.

[12] Buinhas S, Cláudio AP, Carmo MB, García-Alonso J, Fonseca C (2019). Gerontechnology, eds.

[13] Curtis RG, Bartel B, Ferguson T (2021). Improving User Experience of Virtual Health Assistants: Scoping Review. *J Med Internet Res*.

[14] van Calis JFE, Bevelander KE, van der Cruijsen AWC (2023). Toward Inclusive Approaches in the Design, Development, and Implementation of eHealth in the Intellectual Disability Sector: Scoping Review. *J Med Internet Res*.

[15] Jumelle AKL, Ispas I, Gavras A, Fricker SA, Thümmler C (2015). Requirements Engineering for Digital Health, eds.

[16] Danaher J (2018). Toward an Ethics of AI Assistants: an Initial Framework. Philos Technol.

[17] Del Valle JI, Llorca-Albareda JL, Rueda J, Lara F, Deckers J (2024). Ethics of Artificial Intelligence, eds.

[18] van Heijster H, van Calis J, Liebrecht C (2024). The taxonomy of human goals in technology development: supporting needs of long-term care recipients and their caregivers in finding and accessing appropriate care. Research Square.

[19] Goedhart NS, Pittens CACM, Tončinić S (2021). Engaging citizens living in vulnerable circumstances in research: a narrative review using a systematic search. *Res Involv Engagem*.

[20] Fiske A, Prainsack B, Buyx A (2019). Meeting the needs of underserved populations: setting the agenda for more inclusive citizen science of medicine. J Med Ethics.

[21] Kon AA (2009). The role of empirical research in bioethics. Am J Bioeth.

[22] Green J, Thorogood N (2014). Qualitative Methods for Health Research. 4rd Ed.

[23] Braun V, Clarke V (2006). Using thematic analysis in psychology. Qual Res Psychol.

[24] Williams M, Moser T (2019). The art of coding and thematic exploration in qualitative research. *Int Manag Rev*.

[25] Alrassi J, Katsufrakis PJ, Augment C (2021). Critical Human Skills Needed for Patient Care. Acad Med.

[26] Coeckelbergh M (2010). Health Care, Capabilities, and AI Assistive Technologies. Ethic Theory Moral Prac.

[27] Mast MS (2007). On the importance of nonverbal communication in the physician-patient interaction. Patient Educ Couns.

[28] Cohen IG (2023). What Should ChatGPT Mean for Bioethics?. Am J Bioeth.

[29] Eghrari AO (2024). Artificial Intelligence, Medical Knowledge, and Empowering Patients. *Mayo Clinic Proceedings: Digital Health*.

[30] Howell MD (2024). Generative artificial intelligence, patient safety and healthcare quality: a review. BMJ Qual Saf.

[31] Sezgin E (2024). Redefining Virtual Assistants in Health Care: The Future With Large Language Models. *J Med Internet Res*.

[32] McCarthy RL (2008). Ethics and patient privacy. J Am Pharm Assoc (2003).

[33] Blease CJ (2024). Open AI meets open notes: surveillance capitalism, patient privacy and online record access. J Med Ethics.

[34] Stevens M, Kraaijeveld SR, Sharon T (2024). Sphere transgressions: reflecting on the risks of Big Tech expansionism. Inf Commun Soc.

[35] Piasecki S, Chen J (2022). Complying with the GDPR when vulnerable people use smart devices. International Data Privacy Law.

[36] Kerasidou CX, Kerasidou A, Buscher M (2022). Before and beyond trust: reliance in medical AI. J Med Ethics.

[37] Zohny H, McMillan J, King M (2023). Ethics of generative AI. J Med Ethics.

[38] Zohny H, Porsdam Mann S, Earp BD (2024). Generative AI and medical ethics: the state of play. J Med Ethics.

[39] Lister K, Coughlan T, Iniesto F (2020). Accessible conversational user interfaces. considerations for design.

[40] Ali O, Pagliari C, Dalgarno E (2023). How the digital healthcare revolution leaves the most vulnerable behind. J Public Health (Bangkok).

[41] Følstad A, Araujo T, Law EL-C (2021). Future directions for chatbot research: an interdisciplinary research agenda. *Computing*.

[42] Persson H, Åhman H, Yngling AA (2015). Universal design, inclusive design, accessible design, design for all: different concepts—one goal? On the concept of accessibility—historical, methodological and philosophical aspects. Univ Access Inf Soc.

[43] Brandtzaeg PB, Følstad AW, Kompatsiaris I, Cave J, Satsiou A (2017). Internet Science, ed.

