# Supplementary material for: The ethics of using virtual assistants to help people in vulnerable positions access care
Source: J Med Ethics. 2025 Feb 17;52(1):e110464. doi: 10.1136/jme-2024-110464 (PMC12772568; doi:10.1136/jme-2024-110464)
Supplement: online supplemental appendix 1 [file jme-52-1-s001.docx]

Appendix 1. Coding tree with three main themes and seven subthemes.

Complex information

Only simple questions

Complexity of care questions

Independent question formulation

Fewer barriers and shame

Importance of authentic relationships

Referral function

Lower threshold to ask for help

Information source

Speech technology limited

Difficulty with writing and typing

Accessibility virtual assistant

Suitability virtual assistant

Privacy and reliability of information

Previous bad experiences with VA’s

Sensitive topics

Unpersonal and standard messages

Nonverbal communication

Required communicative skills
